# Fingergelenkarthrodesen mittels Cup-and-Cone-Technik: Ein einfaches und sicheres Verfahren

**DOI:** 10.1007/s00113-026-01698-8

**Published:** 2026-03-17

**Authors:** Adrian Sebald, Andreas Arkudas, Raymund E. Horch

**Affiliations:** Plastisch- und Handchirurgische Klinik, Universitätsklinikum Erlangen, Friedrich-Alexander-Universität Erlangen-Nürnberg FAU, Krankenhausstraße 12, 91054 Erlangen, Deutschland

**Keywords:** Sphärische Resektionsarthrodese, Gelenksversteifung, Arthrose, Fingergelenksfusion, Digitalgelenk, Spherical resection arhtrodesis, Bony fusion, Osteoarthritis, Joint satabilization by fusion, Digital articulation

## Abstract

**Hintergrund:**

Fingergelenkarthrosen entstehen häufig infolge von Traumata, degenerativen oder entzündlichen Prozessen. Wenn konservative Maßnahmen und gelenkerhaltende Therapien versagen, stellt die Arthrodese eine bewährte Option zu Schmerzreduktion und Funktionsverbesserung dar. Unter den verschiedenen Techniken hat sich insbesondere die Cup-and-Cone-Methode durch hohe Stabilität und intraoperative Flexibilität bei leichter Durchführbarkeit bewährt. Das Ziel der Arbeit ist es, dies anhand des Patientengutes eines Supramaximalversorgers zu eruieren.

**Material/Methoden:**

Zwischen 2015 und 2025 wurden retrospektiv 201 Fingergelenkarthrodesen bei 168 Patient*innen analysiert. Die Patient*innen waren durchschnittlich 60 Jahre alt, 71 % männlich. Die rechte Hand war bei 54 % der Eingriffe betroffen. Es erfolgte die Erfassung von demografischen Daten, Lokalisation, Ätiologie und Komplikationen. Subgruppenanalysen fokussierten sich auf arthrosebedingte Arthrodesen zur Messung prä- und postoperativer Achsabweichung sowie Gelenkstellung.

**Ergebnisse:**

Die meisten Arthrodesen erfolgten an den PIP-Gelenken (104), gefolgt von DIP- (65) und IP-Gelenken (16). Hauptursache war ein Trauma (48 %), gefolgt von degenerativen Gelenkveränderungen (20 %). Komplikationen traten selten auf; häufigste war die postoperative Infektion. In der Subgruppe degenerativer Arthrosen zeigten sich signifikante postoperative Reduktionen der Achsabweichung (bis zu 25°).

**Schlussfolgerung:**

Fingergelenkarthrodesen sind bei therapierefraktären Beschwerden eine effektive Maßnahme zu Funktionsverbesserung und Beschwerdelinderung. Die Cup-and-Cone-Technik erwies sich als Verfahren mit hoher Erfolgsrate und niedriger Komplikationshäufigkeit. Die individuelle Planung der Gelenkstellung und sorgfältige Technikwahl sind entscheidend für das funktionelle Ergebnis. Zukünftige prospektive Studien sollten die Langzeiteffekte und Patient*innenzufriedenheit weiter untersuchen.

**Graphic abstract:**

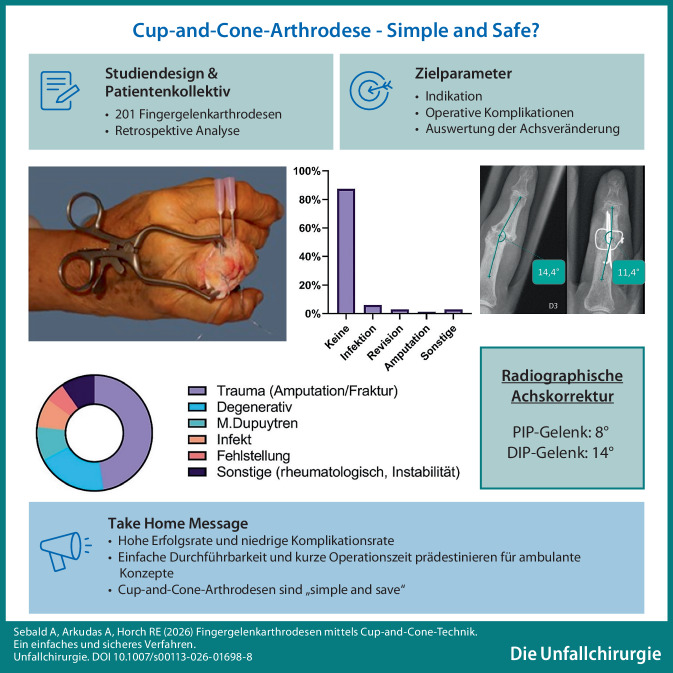

## Abstract (en)

**Background:**

Finger joint arthrosis frequently results from trauma, degenerative or inflammatory causes. When conservative treatment and joint-preserving procedures fail, arthrodesis remains a reliable surgical option to relieve pain and restore hand function. Among the various techniques, the cup and cone method has proven particularly effective due to its large cartilage-free surface area and ease of intraoperative axis correction. The aim of this study is to evaluate this based on the patient cohort of a supramaximum care provider.

**Material and methods:**

A retrospective analysis of 201 finger joint arthrodeses in 168 patients was conducted between 2015 and 2025. Data collected included demographics, joint location, surgical indications and postoperative complications. A subgroup analysis of arthrodesis performed for degenerative osteoarthritis included comparison of preoperative and postoperative axis deviation and flexion angles per joint type.

**Results:**

Patients had a mean age of 60 years and 71% were male. The right hand was affected in 54% of cases. Most arthrodeses involved proximal interphalangeal (PIP) joints (104), followed by distal interphalangeal (DIP, 65) and thumb IP joints (16). The leading indication was posttraumatic arthritis (48%), followed by degeneration (20%). Complication rates were low, with infection being the most common. In degenerative osteoarthritis cases, significant postoperative reductions in axis deviation were observed (up to 25°).

**Conclusion:**

Arthrodesis of the finger joints is an effective measure for improving function and relieving symptoms in treatment-refractory cases. The cup and cone technique has proven to be a procedure with a high success rate and a low complication rate. Individual planning of joint positioning and careful selection of the surgical technique are crucial for the functional outcome. Future prospective studies should further investigate long-term effects and patient satisfaction.

Fingergelenkarthrosen führen häufig zu Schmerzen und Funktionsverlust, bedingt durch Traumata, degenerative oder entzündliche Prozesse. Versagen konservative und gelenkerhaltende Maßnahmen, stellt die Arthrodese ein etabliertes Verfahren dar, um Schmerzfreiheit und Stabilität zu erreichen. Unter den verfügbaren Techniken hat sich die Cup-and-Cone-Methode (sphärische Resektionsarthrodese) durch hohe Primärstabilität, intraoperative Flexibilität und zuverlässige Ergebnisse besonders bewährt.

## Einleitung

Trotz aller Fortschritte in der modernen Handchirurgie bezüglich der Rekonstruktionsmöglichkeiten nach Traumata oder Gelenkinfektionen bzw. septischer Arthritis kommt es nach wie vor nicht selten zu posttraumatischen Arthrosen, besonders in den Fingergelenken. Sowohl bei degenerativ bedingten, posttraumatischen, auf eine systemische Grunderkrankung zurückgehenden oder auch idiopathischen Arthrosen sollte zunächst die gesamte Palette der konservativen Therapiemöglichkeiten ausgeschöpft werden. Bevor die Fingergelenkarthrodese in Erwägung gezogen wird, kann konservativ mittels Schienung, entzündungshemmender sowie schmerzlindernder Pharmakotherapie oder auch interventionell mittels Kortikosteroidinjektionen therapiert werden [1, 2].

*In vielen Fällen kann durch eine geeignete Gelenkendoprothetik oder eine Arthroplastik die Beweglichkeit bei gleichzeitiger Schmerzreduktion weitestgehend erhalten werden *[3, 4]. Zu bedenken ist jedoch, dass die Versorgung mit Endoprothesen ein deutlich höheres Komplikationsrisiko für die Patient*innen bedeutet. Die Literatur zeigt, dass etwa 60 *% operativ revidiert werden müssen. Häufige Probleme sind Osteolysen oder die Migration der Implantate. Das funktionelle Outcome einer Arthrodese erweist sich, bei deutlich geringerem Komplikationsrisiko, als vergleichbar, weswegen die Fingergelenkarthrodese nach wie vor eine wichtige therapeutische Option darstellt. Dies trifft insbesondere am Zeigefinger aufgrund der erhöhten biomechanischen Belastung zu *[5, 6]*.*

Nach dem Ausschöpfen der konservativen Therapie und bei fehlender Indikation zu einer Arthroplastik oder Endoprothetik kann die allgemeine Indikation für Gelenkeinsteifungen an der Hand gestellt werden. Hierfür sollten Schmerzen, Instabilität oder eine nicht mehr korrigierbare Deformität in Verbindung mit einem Verlust der motorischen Kontrolle vorliegen. Arthrodesen an den Fingergelenken sind daher immer dann in das therapeutische Kalkül miteinzubeziehen, wenn alle vorangegangenen Versuche, eine schmerzlose und effektive Beweglichkeit in den Gelenken wiederherzustellen, fehlgeschlagen sind. Durch die Versteifung eines nicht mehr rekonstruierbaren Gelenkes kommt es in der Regel zu einer Verbesserung der Handfunktion sowie zu einer Stabilisierung und zur Schmerzausschaltung. *Dies kann auch in der akuten Traumaversorgung als sinnvolle Versorgungsoption zum Tragen kommen *[7, 8]*.* Am häufigsten wird die Indikation für eine Arthrodese an der Hand für die proximalen und distalen Interphalangealgelenke gestellt, wobei die Ätiologie meist degenerativer, entzündlicher, traumatischer oder posttraumatische Natur ist [9]. *An den Metakarpophalangealgelenken wird die Indikation dagegen nur gestellt, wenn eine Arthroplastik versagt hat, oder wenn kein ausreichend knöchernes Lager für eine Gelenkprothetik besteht* [10, 11].

Obwohl viele unterschiedliche Methoden der internen Fixierung für eine Arthrodese an den Fingergelenken beschrieben sind, hat sich im eigenen Vorgehen insbesondere die Cup-and-Cone-Technik [12] wegen der großen knorpelfreien Oberfläche und der einfachen Möglichkeit der intraoperativen Achsenjustierung bewährt.

Die vorfolgende Studie untersucht die Eignung eines standardisierten einfachen Verfahrens für die Fingergelenkarthrodese anhand des eigenen Krankengutes im Hinblick auf die technische Durchführung und Komplikationen.

## Material und Methoden

Im Rahmen einer retrospektiven Analyse wurden zwischen 2015 und 2025 insgesamt 201 an den Fingern vorgenommene Arthrodesen ausgewertet. Erfasst wurden demografische Daten, die Lokalisation der Gelenkarthrodese, die Ursache des Eingriffs sowie das postoperative Komplikationsprofil. Zur Evaluation des operativen Ergebnisses wurde eine Subgruppenanalyse der aufgrund degenerativer Arthrose durchgeführten Arthrodesen vorgenommen. Dabei erfolgte für die verschiedenen Fingergelenklokalisationen jeweils ein Vergleich der prä- und postoperativen Achsabweichung sowie der postoperativen Flexionsstellung.

Hierfür wurden eine Datenextraktion aus den Akten und dokumentierten Nachuntersuchungen vorgenommen sowie eine Sichtung und Achsvermessung der prä- sowie postoperativen Röntgenbilder in den Achsen anterior-posterior sowie lateral.

Für statistische Analysen und deren grafische Aufbereitung wurde GraphPad Prism, Version 10, benutzt. Vergleiche zwischen zwei Gruppen wurden mittels gepaartem t‑Test durchgeführt. Bei mehreren Gruppen wurde eine gewöhnliche einfaktorielle Varianzanalyse (ANOVA) mit dem Tukey-Mehrfachvergleichstest durchgeführt. Eine statistische Signifikanz wurde bei einem *P*-Wert < 0,05 akzeptiert (NS [*P* ≥ 0,05]; *P < 0,05; **P < 0,01; ***P < 0,001).

Die Auswertung der Daten wurde mit Zustimmung der zuständigen Ethik-Kommission (Antragsnummer: 438_19 B), im Einklang mit nationalem Recht sowie gemäß der aktuellen, überarbeiteten Fassung der Deklaration von Helsinki von 1975 (in der aktuellen, überarbeiteten Fassung) durchgeführt. Die Daten, die die Ergebnisse dieser Studie stützen, sind aus Gründen der Sensibilität nicht frei verfügbar und können auf begründete Anfrage bei der korrespondierenden Autorin/dem korrespondierenden Autor angefordert werden. Die Daten befinden sich in einem Datenspeicher mit kontrollierter Zugriffsbeschränkung.

BioRender, Science Suite Inc, Toronto, Kanada, wurde für die grafische Darstellung benutzt.

## Ergebnisse

Zwischen 2015 und 2025 wurde bei 168 Patient*innen die Versteifung von insgesamt 201 Gelenken vorgenommen. *Die Operationen erfolgten an einem überregionalen Supramaximalversorger mit durchgehendem Replantationsdienst nach Facharztstandard*. Zum Zeitpunkt der Operation lag das durchschnittliche Patient*innenalter bei 60 Jahren (23 bis 98 Jahre). Von den erfassten Patient*innen war bei 71 % das biologische Geschlecht männlich und bei 29 % weiblich.

**Abb. 1:**
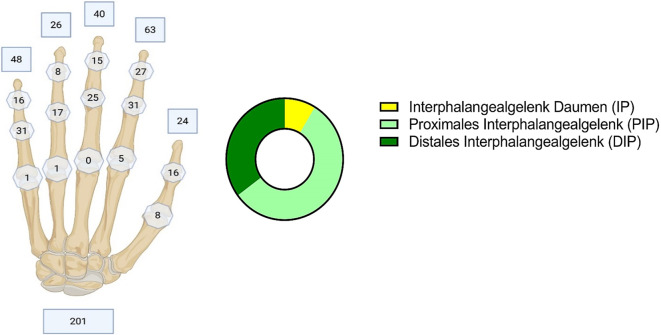
Gelenkverteilung

Bei 54 % wurde die rechte, bei 46 % die linke Seite operiert. Bei Beleuchtung der Arthrodesen nach Gelenktyp offenbart sich eine Verteilung von 16 Operationen auf die Interphalangealgelenke des Daumens (IP), 104 Versteifungen der proximalen Interphalangealgelenke (PIP) und 65 Arthrodesen an den distalen Interphalangealgelenken (DIP) (Abb. 1). Die größere Anzahl der Fälle zeigte sich jeweils bei dem biologisch männlichen Geschlecht (Abb. 1).

**Abb. 2:**
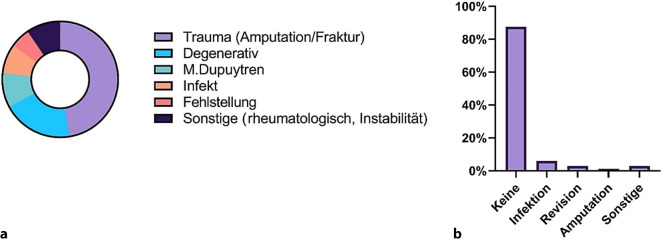
Ätiologie (**a**) und Komplikationen (**b**)

Die in den häufigsten Fällen zur Arthrodese führende Ursache war in 48 % ein akutes Trauma, z. B. eine Fraktur oder Amputation. Degenerative Veränderungen folgten mit 20 %. Morbus Dupuytren erwies sich in 10 % ursächlich, gefolgt von Infekten mit 8 % und Fehlstellungen mit 5 %. In etwa 10 % führten sonstige Ursachen wie etwa rheumatologische Grunderkrankungen oder sekundär posttraumatische Instabilitäten zur Gelenkversteifung. Die Daten ergaben bei den durchgeführten Gelenkarthrodesen in unserem Patientenkollektiv sehr wenige postoperative Komplikationen. 147 Fälle verliefen komplikationslos. Eine Revision war lediglich in 5 Fällen notwendig. In 2 Fällen folgte die Amputation in einem Zweiteingriff. Die postoperative Infektion war die häufigste Komplikation mit 10 Fällen (Abb. 2).

Zur Objektivierung des postoperativen Erfolges wurde eine Subgruppenanalyse der aufgrund von degenerativer Arthrose durchgeführten Gelenkversteifungen vorgenommen. Bei 34 Patient*innen wurden 36 Gelenke versteift. Ein Fall wurde exkludiert, da aufgrund von gravierenden Fehlstellungen die präoperative Röntgendiagnostik nicht zur validen Achsausmessung heranzuziehen war. Ein weiterer Casus ließ sich aufgrund mangelnder Bildgebung nicht für Studienzwecke heranziehen.

**Abb. 3:**
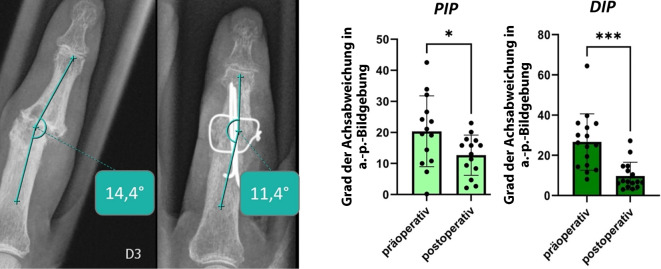
Auswertung der radioulnaren Achskorrektur

*Im a.-p.-Strahlengang der 14 PIP-Gelenke lässt sich postoperativ eine signifikante (p* *<* *0,05) Reduktion der Achsabweichung um durchschnittlich 8 Grad nachweisen. Die 14 ausgewerteten DIP-Gelenke zeigten eine hochsignifikante (p* *<* *0,001) Reduktion der postoperativen Achsdeviation um durchschnittlich 14 Grad (*Abb. 3*).*

**Abb. 4:**
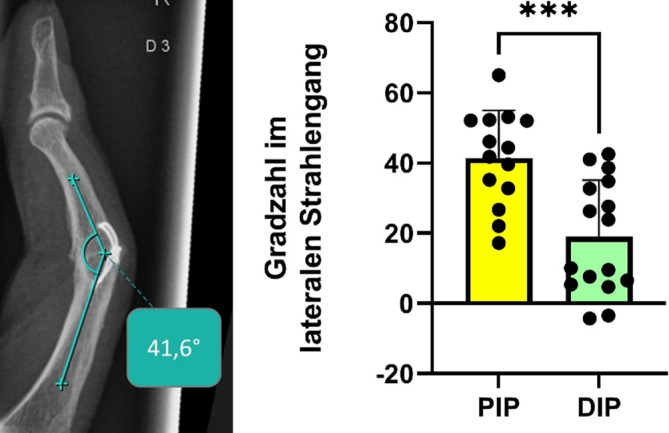
Auswertung der postoperativen Gelenkstellung

*Zur weiteren Objektivierung des postoperativen Erfolgs wurde die Beugung des versteiften Gelenks im lateralen Strahlengang gemessen. Hier zeigte sich anatomiegerecht ein hochsignifikant (p* *<* *0,001) höherer Beugungsgrad im PIP-Gelenk als in den IP- bzw. DIP-Gelenken (*Abb. 4*).*

## Diskussion

**Abb. 5:**
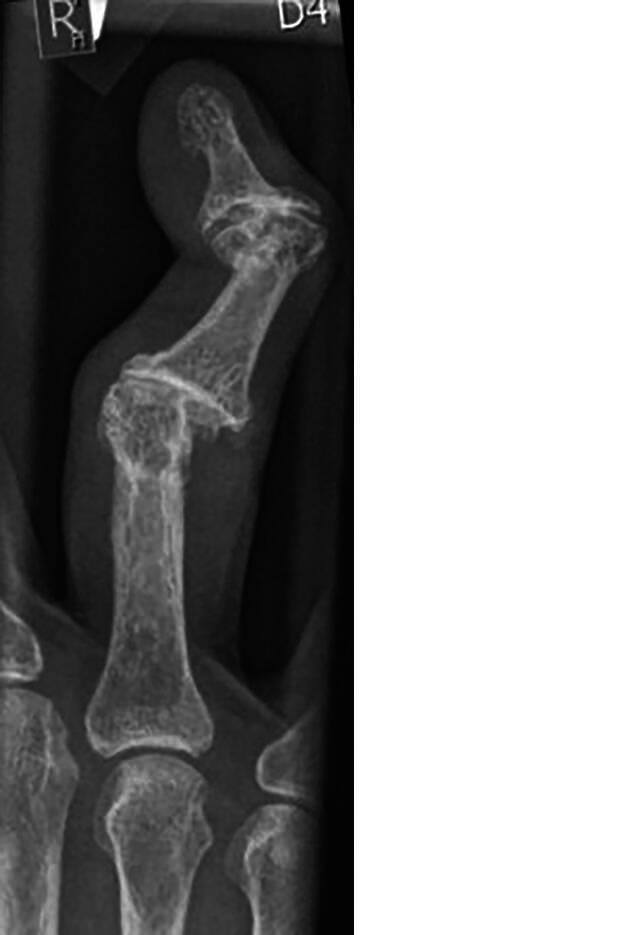
Röntgenaufnahme im a.-p.-Strahlengang eines Ringfingers mit fortgeschrittenen degenerativen Gelenkveränderungen

Abhängig von der zugrunde liegenden Ätiologie existieren unterschiedliche therapeutische Ansätze für therapierefraktäre Gelenkbeschwerden der Hand. Insbesondere degenerative Veränderungen, traumatische Verletzungen oder entzündliche Veränderungen stellen die häufigsten Indikationen für eine operative Arthrodese als ultimatives Mittel der Schmerzbekämpfung dar. Anhand des Patientengutes eines überregionalen Supramaximalversorgers mit durchgehendem Replantationsdienst dominiert die Indikation zur Fingergelenkarthrodese bei traumatisch bedingter Ätiologie. An zweiter Stelle stellen auch in unserem Patientengut degenerative Veränderungen eine häufige Indikation für Arthrodesen. Bei groben Fehlstellungen ist die Funktionsbehinderung offensichtlich (Abb. 5), sodass eine Arthrodese häufig als endgültige Lösung infrage kommt [13].

Auch wenn es allgemein erprobte und empfohlene Positionen und Winkelgrade für eine Arthrodese an den Fingergelenken gibt, sollte dennoch die individuelle Konstellation für die jeweiligen Patient*innen herausgefunden werden. Hierzu empfiehlt sich insbesondere bei kritischen Patient*innen und bei solchen, die mit der Indikationsstellung noch nicht sicher sind bzw. sich die positiven Auswirkungen einer DIP-Gelenk-Versteifung nicht vorstellen können, eine temporäre K‑Draht-Gelenktransfixation in der angedachten Gelenkstellung vor der endgültigen Arthrodese.

Die gebräuchlichen Winkelgrade, wurden auch in unserem Patientengut erreicht (Tab. 1). Dies korrelierte auch mit den von uns gewonnenen klinischen Ergebnissen. Erfahrungsgemäß sollte im Falle einer Arthrodese der Metakarpophalangealgelenke von radial nach ulnar hin in zunehmender Beugestellung vorgegangen werden. Am Zeigefinger sollten 25° sowie dann eine jeweils etwa um 5° Grad gesteigerte Beugung bis zum Kleinfinger angestrebt werden. Wichtig ist es, zu beachten, dass ein Rotationsfehler in den Metakarpophalangealgelenken nur sehr schlecht toleriert wird und daher unbedingt vermieden werden sollte.

**Tab. 1:** Die wesentlichen Indikationen, diagnostischen Maßnahmen und operativen Techniken für Fingergelenkarthrodesen.

Hauptindikation Fingergelenkarthrodesen	Schmerzhafte Fingergelenke, fortgeschrittene arthrotische Veränderungen, Kontraindikationen für einen Gelenkersatz
Präoperative Diagnostik	Röntgenaufnahmen a.-p. und streng seitlich
	Fakultativ: Evaluation der Schmerzfreiheit eines Gelenkes nach intraartikulärer Injektion eines Lokalanästhetikums
Technische Durchführung	K‑Drähte, Cerclagen, Zuggurten, Plattenosteosynthesen, Fixateur externe, Schraubenosteosynthesen

Für die proximalen Interphalangealgelenke gelten als günstige Varianten die Einsteifung in 40° Beugestellung für den Zeigefinger, 45° für den Mittelfinger, 50° für den Ringfinger und 55° für den Kleinfinger. Diese Werte können im Einzelfall entsprechend justiert werden. Für die distalen Interphalangealgelenke wird eine Einsteifung in 0°- bis 5°-Beugestellung empfohlen. Auch wenn in unseren Daten die PIP-Gelenke auf einem signifikant höherem Grad versteift wurden, zeigen unsere DIP-Gelenk-Arthrodesen mehr postoperative Beugung als die empfohlenen 0–5°. Dies mag der Fall sein, da sich möglicherweise im digitalen Zeitalter die Anforderungen wandeln. Ein höherer Beugegrad der DIP-Arthrodese könnte sich für das Bedienen eines Touchscreens als vorteilhaft erweisen.

Der Erfolg der Arthrodese hängt maßgeblich von der guten Aufbereitung der Knochenoberflächen ab, nämlich der Entfernung aller knorpeligen Anteile. Unterschiedliche Faktoren wie die Erfahrung des Chirurgen, Risikokonstellationen etc. wurden für die Komplikationshäufigkeit diskutiert [14]. Es existieren unterschiedlichen Methoden zur Fixierung einer Arthrodese an den Fingergelenken. Anhand der Literatur werden diese in der Regel je nach Knochengröße, patient*innenenspezifischen Besonderheiten und Vorliebe des Chirurgen eingesetzt [15].

**Abb. 6:**
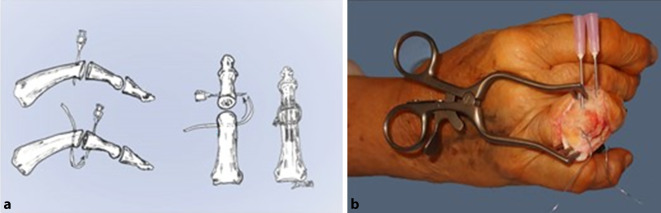
Skizze (mod. n. Horch) einer Arthrodese mittels Cup-and-Cone-Technik (**a**) und intraoperatives Bild der kanülierten Einführhilfe (**b**)

Die wesentlichen Aspekte einer Fingergelenkarthrodese bestehen bekanntlich in der Herstellung eines guten knöchernen Kontaktes, der möglichst viel Spongiosa-Spongiosa-Kontakt erlauben sollte [16]. Ferner sollte nach Möglichkeit keine zu starke Kürzung durch die Resektion von Gelenkflächen stattfinden. Die klassische Osteotomie mit flachen Resektionsebenen ist demnach etwas anspruchsvoller als eine Cup-and-Cone-Technik (Abb. 6). Da besonders für Fingergelenkarthrodesen gilt, dass ein Drehfehler ebenso wie eine zu starke radioulnare Achsenabweichung unbedingt zu vermeiden ist, sollte eine Operationsmethode möglichst einfach zu handhaben sein. Ferner sollte sie intraoperativ möglichst viele Freiheitsgrade bieten, sodass Korrekturmöglichkeiten sowohl in der Rotation als auch der Achsenstellung leicht zu bewerkstelligen sind. Anderst als die klassische Schrägosteotomie bietet sich hierfür die von uns vorgestellte Cup-and-Cone-Methode an. Durch ihre hohe Anfangsstabilität und intraoperative Flexibilität bei leichter Durchführbarkeit kann diese auch von weniger erfahrenen Handchirurgen sicher angewendet werden.

Die Cup-and-Cone-Technik basiert auf der vollständigen Entknorpelung des jeweiligen Gelenkköpfchens entweder mit dem Knochenrongeur von Hand oder mit einem entsprechenden maschinellen Schleifansatz zu einem halbkugelförmigen Kopf und der Schaffung eines entsprechenden konkaven Empfängerlagers im distalen Anteil. Die Schaffung desselben lässt sich z. B. gut mit einem Rosenbohrer durchführen. Unterschiedliche Fixationsmethoden können je nach Vorliebe des Chirurgen eingesetzt werden.

Im Gegensatz hierzu ist bei der klassischen Osteotomie mit der oszillierenden Säge eine sehr sorgfältige präoperative Planung und intraoperative Osteotomie-Schnittführung erforderlich, weil kleinste Abweichungen der Schnittebene zu einer ungünstigen Fingerstellung führen können. Intraoperative Nachkorrekturen der Osteotomieebene führen dann zwangsweise zu einer unnötigen Verkürzung des Fingers.

Die anschließend notwendigen osteosynthetischen Fixierungsmethoden reichen allgemein von einfachen Kirschner-Drähten über Zuggurtungen [17], dynamischen externen Fixierungen [18], Plattenosteosynthesen [19, 20] und intraossären Spongiosa-Spongiosa-Schrauben [21, 22], wie z. B. der Herbert-Schraube [23], speziellen Fusionsimplantaten [24], Klammern [25] und resorbierbaren Fixierungsmethoden [26]. Im eigenen Vorgehen hat sich die Verwendung von Kirschner-Drähten bewährt.

In der Literatur wird gelegentlich diskutiert, ob das Versenken von Kirschner-Drähten unter die Haut mit einem niedrigeren Infektionsrisiko gegenüber überstehenden Drahtenden einhergeht. Bei überstehenden Drahtenden werden eine starke Beeinträchtigung der Patient*innen in Kauf genommen sowie Infektionen bei einer Häufigkeit bis zu 45,5 *% *[27]*. Die wissenschaftliche Datenlage hierzu ist jedoch nicht eindeutig, und bei begrenzten Kollektiven wurden von verschiedenen Autoren keine signifikanten Unterschiede gefunden hinsichtlich des Infektionsrisikos abhängig von der jeweiligen Technik.* [28, 29]*. Da die Kirschner-Drähte für längere Zeit belassen werden müssen, bevorzugen wir im eigenen Vorgehen das Versenken der Kirschner-Drähte unter die Haut.*

*Die Verwendung von Kirschner-Drähten zeigt auch in der Literatur die am häufigsten verwendet Methode, obwohl sich hier eine etwas erhöhte Pseudarthrosenrate zeigt gegenüber Plattenosteosynthesen bei jedoch geringerer Konsolidierungszeit *[30]*. Diesbezüglich muss jedoch gegenübergestellt werden, dass im Gegensatz zum Einsatz von Plattenosteosynthesen der Nutzen von minimalinvasiv eingebrachten Kirschner-Drähten den Vorteil eines minimierten Gewebetraumas hat *[31]*. Eine Zuggurtung mit entsprechenden Stahldrähten erhöht die Festigkeit gegenüber einzelnen Kirschner-Drähten erheblich *[32]. Nach eigenem Vorgehen hat sich die Verwendung von größeren Kanülen als Einführhilfe zum Durchführen der Zuggurtungsdrähte bewährt (Abb. 6). Hierdurch können die Weichteile besser geschont werden.

Bei einer Zuggurtungsosteosynthese wird die gewünschte Winkelstellung eingehalten. Bei der Verwendung einer kanülierten Herbert-Schraube [33] wird das Gelenk im Gegensatz hierzu zwangsläufig bei 0° Flexion transfixiert [13].

*Und ist bewusst, dass diese Studie die allgemeinen Limitierungen aufweist, welche mit retrospektiven Analysen einhergehen.* Dennoch soll dies keinesfalls den praktischen Wert der Arbeit schmälern, sondern vielmehr Ansporn sein, die hier gewonnenen Erkenntnisse weiter zu evaluieren. Allerdings erscheint uns eine prospektive Studie bei traumatischen Gelenkverletzungen auf der anderen Seite ebenfalls problematisch, weil bei diesen Patient*innen naturgemäß keine vorherigen Daten existieren.

## Schlussfolgerung

Unsere Daten hinsichtlich der signifikanten Reduktion der radioulnaren Achsdeviation belegen den hohen Erfolg der Arthrodese durch Cup-and-Cone-Technik mit Kirschner-Drähten und Zuggurtung bzw. Cerclagen bei einer relativ geringen Komplikationsrate. Auch wenn dies nur einen Randeffekt ist, so ist auch aus ökonomischen Aspekten heraus bei einfacherer Durchführbarkeit und kürzerer Operationszeit das Verfahren besonders für die ambulante Behandlung geeignet.

## Data Availability

Data will be made available on reasonable request.
